# Victimisation, poly-victimisation and health-related quality of life among high school students in Vietnam: a cross-sectional survey

**DOI:** 10.1186/s12955-016-0558-8

**Published:** 2016-11-04

**Authors:** Minh T. H. Le, Sara Holton, Huong T. Nguyen, Rory Wolfe, Jane Fisher

**Affiliations:** 1 Jean Hailes Research Unit, School of Public Health and Preventive Medicine, Monash University, 6th Floor, the Alfred Centre, 99 Commercial Road, Melbourne, VIC 3004 Australia; 2 Faculty of Social Sciences, Behaviours and Health Education, Hanoi School of Public Health, 138 Giang Vo street, Ba Dinh District, Hanoi Vietnam; 3 Department of Epidemiology and Preventive Medicine, School of Public Health and Preventive Medicine, Monash University, Level 6, the Alfred Centre, 99 Commercial Road, Melbourne, VIC 3004 Australia

**Keywords:** Adolescents, Violence, Poly-victimisation, Health-related quality of life, Lower-middle income countries

## Abstract

**Background:**

In high and upper-middle income countries poly-victimisation (exposure to multiple forms of victimisation) is associated with worse health-related quality of life (HRQoL) among adolescents. There is a lack of empirical evidence about these associations from low- and lower-middle income countries. The aims of this study were to examine the associations between exposure to 1) individual forms of victimisation and 2) poly-victimisation and the HRQoL of adolescents in Vietnam.

**Method:**

A cross-sectional, anonymously-completed survey of high school students in Hanoi, Vietnam. Lifetime exposure to eight individual forms of victimisation and poly-victimisation were assessed using the Juvenile Victimisation Questionnaire Revised-2 (JVQ R2). Health-related quality of life was assessed using the Duke Health Profile Adolescent Version (DHP-A). Bi-variate analyses and multiple linear regressions were conducted to assess the associations between individual forms of victimisation, poly-victimisation and HRQoL among girls and boys.

**Results:**

In total 1616/1745 students (92.6 %) completed the questionnaire. Adolescent girls had significantly worse HRQoL than boys in all domains, except disability. Different forms of victimisation were associated with different HRQoL domains among girls and boys. Cyber victimisation was the most detrimental to girls’ HRQoL while for boys maltreatment was the most detrimental. Experiences of poly-victimisation were associated with worse HRQoL in physical, mental, social and general health, lower levels of self-esteem and increased levels of anxiety, depression and pain domains among both sexes.

**Conclusions:**

Among Vietnamese adolescents, experiences of individual forms of victimisation were associated with poorer HRQoL in specific domains; the most detrimental forms of victimisation varied for girls and boys. However, it was experiences of poly-victimisation that had the most detrimental impacts on the HRQoL of both sexes. Recognition of violence, including poly-victimisation, is still low in Vietnam. These data indicate that community education, prevention and early intervention programs to reduce violent victimisation and assist adolescents who have experienced it, with attention to gender differences, are needed in Vietnam.

**Electronic supplementary material:**

The online version of this article (doi:10.1186/s12955-016-0558-8) contains supplementary material, which is available to authorized users.

## Background

Conceptualisation and investigation of exposure to and the impact of interpersonal violence against children and adolescents have become more comprehensive in the last decade. Investigations have advanced from those of experiences of individual forms of violence, to those of “multi-type maltreatment” [1–7], which includes physical, psychological and sexual abuse, neglect and witnessing of family violence, progressing to poly-victimisation [8, 9].

Poly-victimisation is defined as exposure to multiple forms of victimisation, not only child maltreatment, but also crime including property victimisation (robbery, theft or intentional vandalism of belongings), physical assault, peer or sibling victimisation, cyber victimisation and witnessing of family or community violence [8, 9]. As well as introducing the concept of poly-victimisation, Finkelhor et al. developed and validated the Juvenile Victimisation Questionnaire (JVQ) to assess poly-victimisation among children and adolescents in the United States [8].

The World Health Organisation defines health as not merely the absence of physical or mental illness, but “a state of complete physical, mental and social well-being” [10]. Health related quality of life (HRQoL) has been widely used as an indicator of “well-being” or quality of life (QoL) [11]. Health-related quality of life moves beyond measurement of physical functioning to include other domains such as mental, social and perceived health domains, self-esteem, body image and autonomy [12–14]. This concept, however, has been criticised for focusing on “a limited set of domains” and not considering other important areas, such as “being productive, having high self-esteem, feeling in control, and having a sense of optimism” [11, 15]. It has also been argued that having poor self-reported health does not necessarily mean low sense of well-being [16]. Similar to other approaches in conceptualising and measuring QoL among children and adolescents, the concept of HRQoL face various theoretical challenges. These include describing the distinction between indicators and determinants of QoL, inclusion of objective versus subjective domains, inclusion of domains important to children from their own perspectives, the rapid development of children, the validity of parent- versus child-reporting of the children’s QoL, and the stability of an individuals’ subjective perceptions of their QoL [11]. Despite these, HRQoL is acknowledged to be helpful in informing effective interventions or treatments, allocation of resources, informing and evaluating policy decisions, and identifying “health-disparities and tracking population trends” [17].

There has been limited investigation of the associations between experiences of violence, including poly-victimisation, and HRQoL among children and adolescents [18–21]. In a systematic review about childhood maltreatment and HRQoL, Weber et al. [18] found only 19 articles which met the inclusion criteria. Most of these studies were conducted among adult survivors of childhood maltreatment; only five studies were representative of more general populations of children and adolescents. The authors noted that among studies which examined multiple types of maltreatment, none consisted of children or adolescents, and an inverse relationship between the number of types of maltreatment experienced and HRQoL was reported.

The available research about exposure to individual forms of violence, poly-victimisation and HRQoL has been conducted mostly in high and upper-middle income countries. Among a nationally representative sample of 6813 German adolescents aged 11–17, Schlack et al. found that victims of multiple incidents of violence during the previous 12 months, experienced a higher burden of psychological problems and impaired HRQoL compared to victims of single episodes of violence [20]. In this study, violence was assessed through the single question: “how often have you been a victim of violence?”; violence was not defined, limiting responses to participants’ subjective interpretations of experiences of violence. The prevalence of violence might thus have been underestimated. Consistent results about the inverse relationship between the number of types of maltreatment experienced and HRQoL were also recorded among a national representative sample of 3202 Swedish 15-year-old students [22]. This study, however, focused on multiple types of child maltreatment and did not investigate poly-victimisation. Among a national representative sample of 7076 Dutch citizens aged 18–64, Afifi et al. reported that exposure to multiple types of childhood maltreatment, was associated with decreased physical and mental HRQoL in adulthood [19]. Chan surveyed 18,341 adolescents aged 15–17 recruited from secondary schools in Hong Kong and mainland China and found that compared to non-victims or victims of fewer forms of victimisation, those who were poly-victimised during their lifetime had significantly lower scores, indicating worse HRQoL on the physical and mental health domains of the commonly used measure the Short-Form-12 (SF-12) [23].

Overall, there is limited evidence about experiences of violence, especially poly-victimisation and their impacts on adolescents in low and lower-middle income countries. In Weber et al.’s systematic review [18], all included studies were conducted in high income countries in Europe, the US, Australia and Kuwait, or upper-middle income countries, including China and Turkey. None of the studies were from a low or lower-middle income country. Another systematic review of evidence about poly-victimisation among children and adolescents from low and lower-middle income settings [24] found no studies of the relationship between poly-victimisation and HRQoL.

Applying an ecological model [25] to the context of low- and lower-middle income countries, it is apparent that multiple determinants of health at the individual, relationship, community and society level are more prevalent and may have a much larger impact in these settings, compared to in high- and upper-middle income countries. Such determinants include prevalence of disease, interpersonal violence, gender inequality, low educational attainment, poverty, unemployment, lack of resources, overcrowded housing, and inaccessible and under resourced health care systems. Cultural values and beliefs may also play an important role in an individual’s health and well-being in these settings. It is unknown how common victimisation is among adolescents, how individual forms of victimisation affect adolescents’ HRQoL, and whether poly-victimisation has an independent impact on the quality of life of adolescents living in low and middle-income countries. It may be expected that children and adolescents in resource-constrained countries, especially those exposed to victimisation and poly-victimisation, will have poorer HRQoL compared to those in more affluent settings. However, there has been limited empirical evidence from these settings to ascertain this [24].

Vietnam is a South East Asian lower-middle income country and there is minimal research evidence about health-related quality of life among Vietnamese children and adolescents. Examining this construct among adolescents in Southern Vietnam, Vo et al. [26] found that being a girl or living in a single-parent-family was associated with worse HRQoL compared to being male or living with both parents. Although experiences of violence among children and adolescents have been investigated in Vietnam, the relationship between these exposures, especially to poly-victimisation, and health-related quality of life has not been investigated specifically [4, 27]. It is not known if exposure to different forms of victimisation is associated with poorer HRQoL; how individual forms of victimisation interact when examined simultaneously; and if accrued experiences of poly-victimisation are associated with poorer HRQoL among young people in Vietnam. Answers to these questions will help identify priority areas for prevention, inform future interventions, policy and practice, and assist with decision making about resource allocation in this lower-middle income country.

The aims of this study were to examine the relationships between: 1) individual forms of violent victimisation and 2) poly-victimisation and HRQoL among adolescents in Vietnam.

## Method

A cross-sectional, anonymously-completed, self-administered survey was conducted among Vietnamese high school students from October 2013 to January 2014. The methods of this study have been reported elsewhere in more detail [27] but are summarised below.

### Setting

The study was conducted in Hanoi, the capital city of Vietnam, which has a population of six million people and includes both urban and rural areas [28]. The majority of residents aged ≥ 15 years in Hanoi (98.2 %) are able to read and write; which is slightly higher than the national literacy rate (94.8 %) [29].

### Participants

Inclusion criteria were that participants were female and male students from randomly selected grade 10–12 classes from four public high schools, four private high schools and two centres for continuing education in a rural and an urban district of Hanoi. The schools and centres were purposively selected to reflect the three main types of high school in Vietnam. Students aged less than 18 years old whose parents did not give permission for them to participate were excluded from the study.

### Materials

The questionnaire comprised 86 items, including study-specific questions and standardised instruments. Study-specific questions were used to assess demographic characteristics, parental alcohol or drug abuse, perceptions about academic workload, satisfaction with academic results and permanent disability or chronic disease. Standardised instruments included 14-items developed by Turner and Butler [30] to assess experiences of adverse life events, including lifetime experiences of fire or natural disasters, severe accidents or illnesses, severe accidents or illnesses of family members, homelessness, parental unemployment, parental incarceration, and death of close friends or family members. Instruments to assess experiences of poly-victimisation and health-related quality of life, which were the exposure and outcome of interest of this paper are described in more detail below.

Lifetime exposure to poly-victimisation was assessed using an “enhanced version” [31] of the Juvenile Victimisation Questionnaire Revision-2 (JVQ R2) Youth Self-reported Screener [32]. The original JVQ comprises 34 yes/no questions and a total score is calculated as the sum of all items. Poly-victimisation can be assessed using this total score as a continuous [33] or categorical variable [34]. Finkelhor et al. categorised scores into two groups at a cut-off point of four or more on the total score if the period of exposure was the previous year [34] and the top 10 % of scores if lifetime experiences were assessed [35]. Various cut-off scores, ranging from four or more [23, 34] to 11 or more were reported in subsequent studies [31, 36].

In the JVQ R2 Youth Self-reported Screener used in this study, there were 37 fixed-choice yes/no items in this instrument, assessing 37 forms of victimisation, including robbery, theft, property vandalism, physical assault, child maltreatment, peer or sibling victimisation, sexual victimisation, witnessing of family or community violence and cyber victimisation. The JVQ R2 was translated into Vietnamese, field-tested and piloted among adolescents in the target age range before administration in the main survey [36]. This scale has been shown to perform well in this sample, with a Cronbach alpha of 0.855.

Health-related quality of life (HRQoL) was assessed using the Duke Health Profile-Adolescent Version (DHP-A) [26, 37]. The DHP-A measures ten HRQoL dimensions, including six “health” domains: physical, mental, social, perceived and general health and self-esteem; and four “dysfunction” domains: anxiety, depression, pain and disability [26, 37, 38]. Details of the items used to create these subscales are presented in Additional file 1: Table S1. General health was calculated as the average of physical, mental and social health scores.

According to the original scoring of the Duke Health Profile, the scores of the six health domains range from 0 to 100 with higher scores indicating better HRQoL [38]. The scores of the four dysfunction domains range from 0 to 100 with higher scores indicating poorer HRQoL [38]. The DHP-A has been formally validated among a sample of Vietnamese adolescents and shown to be reliable and suitable for use among this population [26, 37].

### Procedure

An information package about the study was distributed to students and their parents or other caregivers a week before the survey was administered. Contact details of the researchers were provided to enable questions or concerns to be addressed. It included a form for parents to complete and return if they did not want their children to participate.

On the day of administration, a questionnaire and an envelope were distributed to each student in the selected classes. Students were asked to sit at the two ends of the table to avoid seeing each other’s responses and use the envelope to cover their answers if needed. Those who did not want to participate or whose parents did not give permission for them to participate were asked to leave the questionnaire blank, place it and the parent-signed withdrawal form into the envelope and complete other academic activities quietly. Those who agreed to participate were given instructions on how to complete the questionnaire. The survey was conducted during a normal class session. All students were asked to place the questionnaire into the envelope, seal it and return it to the researchers at the end of the class session.

### Data management and analysis

For each of the 37 questions included in the JVQ R2, a “yes” response was coded as 1 and “no” as 0. Among the eight victimisation modules, six included aggregated experiences of any property victimisation, maltreatment, peer or sibling victimisation, sexual victimisation, witnessing of family violence, and witnessing of community violence were created following the scoring methods of the JVQ-R2 [31, 32, 39] and a previous publication [31]. In addition to these suggested scoring methods, users of the JVQ R2 are encouraged to create their own scoring to suit the purposes of their research [39]. We created a physical assault module, which was based on five items, indicating exposure to any assault with or without weapons, attempted assault, kidnapping or attack due to discrimination and did not include items which were already in the maltreatment and peer or sibling victimisation modules. This was to avoid overlap between the modules. The scoring of these seven modules indicated whether the adolescent had experienced any of these forms of victimisation (“yes/no”). The eighth module, which was experience of cyber victimisation, was determined based on a supplemental item of the JVQ-R2 (“Has anyone ever used the Internet to bother or harass you or to spread mean words or pictures about you?”). A ‘yes’ response to this question was categorised as indicating exposure to cyber victimisation and a “no” non-exposure. The modules and their items are outlined in Additional file 2: Table S2. Correlations between the eight victimisation modules were also examined.

A total victimisation score was calculated for each participant and was the sum of their responses to the 37 questions of the JVQ R2 – this score ranged from 0 to 37. A categorical variable of victimisation was created based on the total victimisation score, in which those who scored 0 were categorised as “non-victims”, those who scored from 1 to 10 (inclusive) as “victims of up to 10 forms” and those who scored more than 10 as “poly-victims”. This cut-off score was consistent with our previous research [36] and enabled comparison with the results of others [31].

In the validation study of the DHP-A among Vietnamese adolescents, scoring of the four dysfunction domains were modified so that higher scores indicate better HRQoL [26, 37]. However, in this study, the original scoring of the Duke Health Profile was used for clarity and to permit international comparisons: higher scores indicate more symptoms of anxiety, depression, pain or disability.

Means and standard deviations of scores on each of the ten dimensions on the DHP-A, including physical, mental, social, perceived and general health, anxiety, depression, pain and disability, were calculated. Comparisons were made between groups on the basis of sex and categories of poly-victimisation.

Questionnaires with more than a third of questions unanswered were excluded from the analyses as they were considered to be missing too much data. Questionnaires missing responses to less than a third of the total questions were managed using multiple imputation. All analyses were conducted using STATA 12.0 and subsequent analyses were performed on imputed data. Bivariate analyses were conducted to examine the relationships between each victimisation module of the JVQ-R2 and the ten domain scores of the DHP-A. In the next step, multiple linear regressions were performed where all eight victimisation modules were entered simultaneously in each module. These eight victimisation modules were then substituted by poly-victimisation (using the JVQ R2 total score) in subsequent multiple linear regressions. All regressions were conducted separately for girls and boys. All models were adjusted for potential confounding factors, including age, rural/urban residence, family composition, socio-economic status, presence of a chronic disease or disability, school type and number of traumatic events experienced. A significance level of 0.05 was used.

## Results

A total of 1616/ 1745 eligible students (92.5 %) returned a completed questionnaire. The 129 students who did not complete the questionnaire included 120 who were absent on the day of the survey, seven who elected not to participate and two whose parents did not permit participation. Ten students missed more than a third of the questions and their data were not included in the analyses; the final sample consisted of 1606 records. Overall, 45.6 % of the sample were girls and nearly half (49.1 %) lived in a rural area. The students had a mean age of 16.5 ± 1.0 years; range 14.9–25.0 years.

Detailed data about the prevalence of individual forms of victimisation and poly-victimisation in this cohort have already been reported [36]. In summary, 78.5 % of the respondents reported lifetime experiences of physical assault; 63.9 % of property victimisation; 64.8 % reported child maltreatment; 60.2 % reported peer or sibling victimisation; 26.8 % reported sexual victimisation; 57.1 % had witnessed family violence and 75.9 % community violence; and 28.3 % had experienced cyber victimisation. More than 94 % of the students had experienced at least one form of victimisation and more than a third (31.1 %) had experienced more than 10 forms of victimisation during their lifetime.

Prevalence of victimisation and poly-victimisation among girls and boys are presented separately in Table 1. Overall, girls were more likely to experience property victimisation, maltreatment, sexual victimisation, witnessing of family violence and community violence than boys. They were also more likely to experience poly-victimisation. Boys were more likely than girls to report experiencing physical assault. There was no difference between girls and boys in terms of their experiences of peer/sibling victimisation and cyber victimisation.

**Table 1:** Prevalence of different forms of victimisation and poly-victimisation among female and male Vietnamese high school students

Forms of victimisation	Girls	Boys
	n (%)	n (%)
Any property victimisation*	483 (67.2)	528 (61.3)
Any physical assault*	377 (52.7)	521 (60.7)
Any maltreatment**	508 (70.3)	517 (60.2)
Any peer/sibling victimisation	420 (58.5)	521 (61.6)
Any sexual victimisation**	251 (35.0)	167 (19.7)
Any witnessing of family violence**	493 (68.7)	395 (47.0)
Any witnessing of community violence**	580 (80.9)	611 (71.8)
Any cyber victimisation	202 (28.2)	241 (28.3)
Poly-victimisation*		
Non-victims	24 (3.7)	55 (7.3)
Victims of 1–10 forms	413 (63.2)	478 (63.3)
Poly-victims	216 (33.1)	222 (29.4)

*p < 0.05, **p < 0.001 for comparison between girls and boys

Correlation co-efficients between eight forms of victimisation investigated in this study are presented in Additional file 3: Table S3. Overall, there were weak correlations between all forms of victimisation.

### Health-related quality of life characteristics

Means and standard deviations (SDs) of scores on the ten dimensions of the DHP-A are presented in Table 2. In general, compared to boys, adolescent girls had significantly worse HRQoL on all dimensions of the DHP-A, except for the disability domain.

**Table 2:** Summary statistics for 10 domains of the DHP-A among Vietnamese high school students

Variables	Girls			Boys			Total sample		
	Mean ± SD		*P*- value	Mean ± SD		*P*- value	Mean ± SD		*P*- value
	This sample	Comparison^a,b^		This sample	Comparison^a,b^		This sample	Comparison^a,b^	
Physical health**	63.5 ± 19.8	80	<0.001	74.3 ± 18.3	86	<0.001	69.3 ± 19.7	83	<0.001
Mental health**	52.8 ± 21.3	71	<0.001	63.1 ± 21.9	75	<0.001	58.4 ± 22.2	73	<0.001
Social health**	64.0 ± 18.9	56	<0.001	69.2 ± 18.2	59	<0.001	66.8 ± 18.7	58	<0.001
Perceived health**	63.3 ± 34.1	58	<0.001	68.8 ± 32.7	62	<0.001	66.3 ± 33.4	60	<0.001
General health**	60.1 ± 14.6	70	<0.001	69.0 ± 14.4	73	<0.001	64.9 ± 15.2	72	<0.001
Self-esteem**	62.6 ± 20.3	66	<0.001	71.9 ± 18.7	69	<0.001	67.6 ± 20.0	68	0.42
Anxiety**	40.0 ± 18.1			32.2 ± 19.0			35.8 ± 18.9		
Depression**	45.4 ± 21.1			35.3 ± 22.0			40.0 ± 22.2		
Pain**	40.1 ± 31.5			31.3 ± 30.7			35.4 ± 31.4		
Disability	91.5 ± 21.9			89.5 ± 25.7			90.4 ± 24.0		

*SD* standard deviation

**p < 0.001 in t-tests for differences between girls and boys

^a^The comparison was from Hanh et al.’s study among 1408 adolescents aged 12–19 years in Vietnam [26]

^b^Comparisons for anxiety, depression, pain and disability domains were not possible due to differences in creating these domains between the two studies

**Fig. 1:**
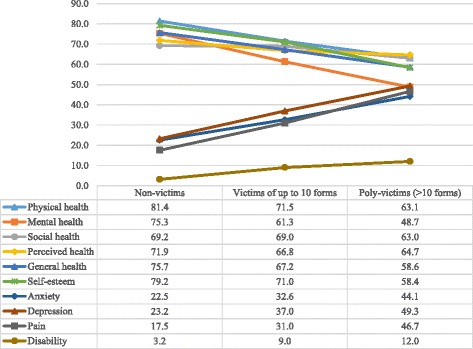
Mean scores of ten DHP-A domain scores (range 0–100) by victimisation categories. *P* < 0.05 for all between-group comparisons among non-victims, victims of up to 10 forms and poly-victims

Adolescents who experienced any form of victimisation, but especially poly-victimisation, reported lower health-related quality of life (see Fig. 1). The mean scores of all six DHP-A health domains were significantly lower for victims of up to 10 forms of victimisation and for poly-victims compared to non-victims. The mean scores of the four DHP-A dysfunction domains, on the other hand, were significantly higher (showing more symptoms of mental health problems, pain and disability) for victims of up to 10 forms and poly-victims, compared to non-victims.

### Bi-variate associations between different forms of victimisation, poly-victimisation and health-related quality of life

For the six DHP-A health domains, physical health, mental health, perceived health, social health, general health and self-esteem, lower scores indicate poorer HRQoL. Results from the bi-variate analyses showed that girls and boys who were victims of individual forms of victimisation had significantly worse HRQoL compared to non-victims. All victimisation modules, except for witnessing of community violence, were associated with significantly lower scores on some of the six health domains of the DHP-A among both sexes (see Additional file 4: Table S4). Witnessing of community violence was not associated with any of these domains.

For the four DHP-A dysfunction domains, anxiety, depression, pain and disability, higher scores indicate more dysfunctional symptoms and worse HRQoL. Results of the bi-variate analyses showed that for both girls and boys there were significant associations between seven out of the eight victimisation modules examined, except for witnessing of community violence, and higher scores on anxiety and depression (see Additional file 5: Table S5). For the pain domain, property victimisation, child maltreatment, peer/sibling victimisation, witnessing of community violence and cyber victimisation were associated with higher pain scores among girls; almost all forms of victimisation, apart from witnessing of community violence, were associated higher pain scores among boys. For the disability domain, only cyber victimisation was associated with higher scores among girls and none of the victimisation modules showed a significant association with this domain among boys.

### Multivariate associations between different forms of victimisation and health-related quality of life

The multiple linear regressions showed that among both girls and boys, when all eight forms of victimisation were taken into account, only some were associated with significantly poorer health-related quality of life (see Tables 3, 4 and 5). Specifically, for girls, property victimisation, maltreatment, sexual victimisation, witnessing of family violence and cyber victimisation were associated with significantly poorer HRQoL in at least one of the ten domains, except perceived health; witnessing of community violence was associated with better general health, while physical assault and peer/sibling victimisation were not associated with any domains. For boys, almost all forms of victimisation, except for property victimisation, had significant associations with at least one of the ten DHP domains, except disability.

**Table 3:** Relationships between different forms of victimisation and six health domains of the DHP-A among Vietnamese high school students - multiple linear regressions^a,b^ (all forms of victimisation were entered simultaneously into the same models)

	Physical health		Mental health		Social health		Perceived health		General health		Self esteem	
	Adjusted β (95 % CI)		Adjusted β (95 % CI)		Adjusted β (95 % CI)		Adjusted β (95 % CI)		Adjusted β (95 % CI)		Adjusted β (95 % CI)	
	Girls	Boys	Girls	Boys	Girls	Boys	Girls	Boys	Girls	Boys	Girls	Boys
Any property victimisation (yes vs no)	**−6.2 (−9.4; −3.0)**	−1.7 (−4.6; 1.1)	−1.2 (−4.7; 2.2)	−2.7 (−6.0; 0.6)	−0.1 (−3.3; 3.2)	−1.6 (−4.6; 1.3)	−4.2 (−10.1; 1.7)	−1.3 (−6.8; 4.2)	**−2.5 (−4.8; −0.2)**	−2.0 (−4.1; 0.1)	−0.3 (−3.6; 3.0)	−2.7 (−5.6; 0.1)
Any physical assault (yes vs no)	−0.2 (−3.3; 3.0)	−1.3 (−4.2; 1.7)	−2.0 (−5.4; 1.4)	**−3.8 (−7.1; −0.4)**	−1.3 (−4.5; 2.0)	**−3.6 (−6.7; −0.6)**	2.2 (−3.6; 8.0)	−0.5 (−6.1; 5.1)	−1.1 (−3.4; 1.1)	**−2.9 (−5.1; −0.7)**	−1.3 (−4.6; 1.9)	−2.6 (−5.5; 0.4)
Any child maltreatment (yes vs no)	−0.7 (−4.1; 2.6)	**−5.0 (−7.8; −2.2)**	**−4.0 (−7.7; −0.3)**	**−4.5 (−7.7; −1.3)**	**−5.3 (−8.8; −1.9)**	−2.7 (−5.5; 0.2)	2.6 (−3.6; 8.8)	−0.9 (−6.2; 4.3)	**−3.4 (−5.8; −0.9)**	**−4.1 (−6.1; −2.0)**	**−5.7 (−9.1; −2.2)**	**−4.8 (−7.5; −2.0)**
Any peer or sibling victimisation (yes vs no)	−0.1 (−3.2; 3.0)	−1.8 (−4.7; 1.1)	−0.2 (−3.6; 3.3)	**−4.8 (−8.2; −1.5)**	−0.6 (−3.8; 2.6)	−1.6 (−4.7; 1.4)	5.0 (−0.7; 10.8)	**−5.6 (−11.1; −0.1)**	−0.3 (−2.5; 2.0)	**−2.8 (−4.9; −0.6)**	−0.3 (−3.6; 2.9)	**−3.8 (−6.8; −0.9)**
Any sexual victimisation (yes vs no)	0.2 (−2.9; 3.3)	−0.4 (−3.7; 2.9)	−1.1 (−4.5; 2.3)	−2.9 (−6.7; 1.0)	0.2 (−3.0; 3.5)	−2.0 (−5.5; 1.4)	−2.9 (−8.6; 2.9)	4.9 (−1.4; 11.2)	−0.2 (−2.5; 2.0)	−1.8 (−4.3; 0.7)	−0.4 (−3.6; 2.9)	−2.3 (−5.6; 1.0)
Any witnessing of family violence (yes vs no)	−1.8 (−5.1; 1.4)	−0.7 (−3.5; 2.1)	−3.0 (−6.6; 0.6)	−1.5 (−4.8; 1.7)	**−3.5 (−6.9; −0.02)**	−2.4 (−5.3; 0.5)	−2.2 (−8.4; 4.0)	0.7 (−4.6; 6.0)	**−2.8 (−5.2; −0.4)**	−1.6 (−3.7; 0.5)	**−5.9 (−9.4; −2.5)**	**−3.2 (−6.1; −0.4)**
Any witnessing of community violence (yes vs no)	1.8 (−2.1; 5.6)	1.8 (−1.3; 4.8)	1.6 (−2.6; 5.9)	**3.8 (0.2; 7.4)**	5.9 (2.0; 9.9)	2.8 (−0.4; 6.0)	3.7 (−3.3; 10.8)	2.2 (−3.7; 8.1)	**3.1 (0.3; 5.9)**	**2.8 (0.5; 5.1)**	0.5 (−3.5; 4.5)	2.6 (−0.5; 5.8)
Any cyber victimisation (yes vs no)	**−6.9 (−10.2; −3.7)**	−2.3 (−5.2; 0.7)	**−7.2 (−10.7; −3.7)**	**−4.7 (−8.1; −1.4)**	−2.5 (−5.8; 0.8)	0.7 (−2.3; 3.8)	−4.6 (−10.5; 1.3)	**−2.3 (−7.8; 3.2)**	**−5.6 (−7.9; −3.2)**	−2.1 (−4.3; 0.1)	**−3.8 (−7.1; −0.4)**	−2.6 (−5.6; 0.3)

^a^Model adjusted for age, rural/ urban residence, family composition, socio-economic status, presence of a chronic disease or disability, school type and number of adverse life events experienced

^b^Significant results are in bold

**Table 4:** Relationships between different forms of victimisation and four dysfunction domains of the DHP-A among Vietnamese high school students - multiple linear regressions^a,b^ (all forms of victimisation were entered simultaneously into the same models)

	Anxiety		Depression		Pain		Disability	
	Adjusted β (95 % CI)		Adjusted β (95 % CI)		Adjusted β (95 % CI)		Adjusted β (95 % CI)	
	Girls	Boys	Girls	Boys	Girls	Boys	Girls	Boys
Any property victimisation (yes vs no)	2.6 (−0.3; 5.5)	2.8 (−0.1; 5.6)	2.9 (−0.5; 6.3)	1.6 (−1.7; 4.9)	**8.1 (2.8; 13.3)**	1.4 (−3.4; 6.1)	2.7 (−1.0; 6.5)	−2.7 (−6.8; 1.5)
Any physical assault (yes vs no)	1.8 (−1.0; 4.7)	**3.7 (0.8; 6.7)**	2.8 (−0.6; 6.1)	**4.3 (0.9; 7.7)**	0.2 (−5.0; 5.3)	1.8 (−3.1; 6.8)	−0.5 (−4.2; 3.2)	3.7 (−0.6; 8.0)
Any child maltreatment (yes vs no)	**3.6 (0.5; 6.6)**	**4.4 (1.6; 7.2)**	2.1 (−1.5; 5.7)	**4.8 (1.6; 8.0)**	3.2 (−2.3; 8.7)	**6.1 (1.4; 10.8)**	−3.8 (−7.8; 0.1)	2.3 (−1.8; 6.4)
Any peer or sibling victimisation (yes vs no)	1.3 (−1.6; 4.1)	**3.4 (0.4; 6.3)**	−0.3 (−3.7; 3.0)	**4.0 (0.6; 7.4)**	1.9 (−3.2; 7.0)	3.1 (−1.9; 8.0)	1.0 (−2.6; 4.7)	2.0 (−2.4; 6.3)
Any sexual victimisation (yes vs no)	1.1 (−1.7; 4.0)	**3.5 (0.2; 6.8)**	**3.4 (0.02; 6.8)**	3.2 (−0.7; 7.1)	−1.3 (−6.4; 3.8)	3.3 (−2.3; 8.8)	0.4 (−3.2; 4.1)	−3.1 (−8.0; 1.8)
Any witnessing of family violence (yes vs no)	2.1 (−0.9; 5.1)	1.1 (−1.7; 4.0)	2.5 (−1.1; 6.0)	0.9 (−2.3; 4.2)	1.2 (−4.3; 6.7)	1.9 (−2.8; 6.6)	−1.2 (−5.1; 2.7)	−0.9 (−5.0; 3.2)
Any witnessing of community violence (yes vs no)	−3.2 (−6.7; 0.3)	−2.2 (−5.3; 1.0)	−3.5 (−7.7; 0.6)	**−3.9 (−7.5; −0.2)**	3.9 (−2.4; 10.3)	−0.2 (−5.5; 5.1)	−0.1 (−4.7; 4.4)	−1.9 (−6.4; 2.7)
Any cyber victimisation (yes vs no)	**5.6 (2.7; 8.5)**	2.5 (−0.4; 5.4)	**6.6 (3.1; 10.0)**	**5.2 (1.8; 8.6)**	**5.8 (0.4; 11.2)**	3.8 (−1.2; 8.8)	**4.2 (0.4; 7.8)**	−0.1 (−4.4; 4.2)

^a^Model adjusted for age, rural/ urban residence, family composition, socio-economic status, presence of a chronic disease or disability, school type and number of adverse life events experienced

^b^Significant results are in bold

**Table 5:** Summary of the associations between different forms of victimisation, poly-victimisation and health-related quality of life among Vietnamese high school students

	Physical health^a^	Mental health^a^	Social health^a^	Perceived health^a^	General health^a^	Self-esteem^a^	Anxiety^b^	Depression^b^	Pain^b^	Disability^b^
Girls										
Property victimisation	-				-				+	
Physical assault										
Maltreatment		-	-		-	-	+			
Peer/sibling victimisation										
Sexual victimisation								+		
Witnessing of family violence			-		-	-				
Witnessing of community violence					+					
Cyber victimisation	-	-			-	-	+	+	+	+
Poly-victimisation	-	-	-		-	-	+	+	+	
Boys										
Property victimisation										
Physical assault		-	-		-		+	+		
Maltreatment	-	-			-	-	+	+	+	
Peer/sibling victimisation		-		-	-	-	+	+		
Sexual victimisation							+			
Witnessing of family violence						-				
Witnessing of community violence		+			+					
Cyber victimisation		-		-						
Poly-victimisation	-	-	-		-	-	+	+	+	

^a^For physical, mental, social, perceived and general health and self-esteem, a “-” indicates a significant negative association, i.e. associated with poorer health-related quality of life (HRQoL); a “+” indicates a significant positive association, i.e. associated with better HRQoL

^b^For anxiety, depression, pain and disability, a “-” indicates a significant negative association, i.e. associated with less dysfunctional symptoms or better HRQoL; a “+” indicates a significant positive association, i.e. associated with more dysfunctional symptoms or poorer HRQoL

Different forms of victimisation were associated with different domains of HRQoL (see Table 5). For example, property victimisation was associated with poorer physical health and an increased risk of pain among girls; but not associated with any of the domains for boys; physical assault was associated with poorer mental and social health and increased risks of anxiety and depression among boys, but not with any of the domains for girls. Witnessing of community violence, on the other hand, was associated with better mental health among boys, and better general health among both sexes.

The form of victimisation which was the most detrimental to HRQoL (showing significant associations with the highest number of HRQoL domains) differed for girls and boys. For girls, cyber victimisation appeared to be the most detrimental form of victimisation and was significantly associated with eight out of 10 HRQoL domains, including physical, mental, general health, self-esteem, anxiety, depression, pain and disability. Maltreatment was the second most detrimental form of victimisation for girls’ HRQoL and was associated with five domains (mental, social and general health, self-esteem and anxiety). For boys, maltreatment appeared to be the most detrimental form of victimisation and was associated with seven domains (physical, mental and general health, self-esteem, anxiety, depression and pain), and peer/sibling victimisation was the second most detrimental, showing significant associations with six domains (mental, perceived and general health, self-esteem, anxiety and depression).

### Multivariate associations between poly-victimisation and health-related quality of life

Multivariate linear regressions revealed consistent associations between poly-victimisation and poorer HRQol among both girls and boys in this sample (see Tables 6 and 7). Particularly, poly-victimisation was significantly associated with poorer physical, mental, social and general health, lower levels of self-esteem and increased risks of anxiety, depression and pain for both sexes. There was no association between poly-victimisation and perceived health or disability.

**Table 6:** Relationships between poly-victimisation and six health domains of the DHP-A among Vietnamese high school students - multiple linear regressions^a,b^

	Physical health		Mental health		Social health		Perceived health		General health		Self esteem	
	Adjusted β (95 % CI)		Adjusted β (95 % CI)		Adjusted β (95 % CI)		Adjusted β (95 % CI)		Adjusted β (95 % CI)		Adjusted β (95 % CI)	
	Girls	Boys	Girls	Boys	Girls	Boys	Girls	Boys	Girls	Boys	Girls	Boys
Poly-victimisation (Total JVQ R2 score)	**−0.7 (−1.0; −0.4)**	**−0.6 (−0.8; −0.3)**	**−1.0 (−1.3; −0.7)**	**−1.1 (−1.4;-0.8)**	**−0.5 (−0.8; −0.2)**	**−0.6 (−0.8; −0.3)**	0.01 (−0.5; 0.5)	−0.3 (−0.8; 0.2)	**−0.8 (−1.0; −0.5)**	**−0.7 (−0.9; −0.6)**	**−1.0 (−1.3; −0.7)**	**−1.0 (−1.3; −0.8)**

^a^Model adjusted for age, rural/ urban residence, family composition, socio-economic status, presence of a chronic disease or disability, school type and number of adverse life events experienced

^b^Significant results are in bold

**Table 7:** Relationships between poly-victimisation and four dysfunction domains of the DHP-A among Vietnamese high school students - multiple linear regressions^a,b^

	Anxiety		Depression		Pain		Disability	
	Adjusted β (95 % CI)		Adjusted β (95 % CI)		Adjusted β (95 % CI)		Adjusted β (95 % CI)	
	Girls	Boys	Girls	Boys	Girls	Boys	Girls	Boys
Poly-victimisation (Total JVQ R2 score)	**0.9 (0.6; 1.2)**	**1.0 (0.7; 1.2)**	**1.0 (0.7; 1.3)**	**1.0 (0.7; 1.3)**	**1.2 (0.8; 1.7)**	**1.1 (0.7; 1.6)**	0.1 (−0.2; 0.4)	0.1 (−0.3; 0.5)

^a^Model adjusted for age, rural/ urban residence, family composition, socio-economic status, presence of a chronic disease or disability, school type and number of adverse life events experienced

^b^Significant results are in bold

Overall, poly-victimisation appeared to be more detrimental to adolescents’ HRQoL compared to single specific forms of victimisation for both girls and boys. For example, the beta co-efficient of 1.1 for DHP mental health among males indicates that adjusting for other factors, male poly-victims (whose JVQ R2 scores were 11 or more) had a mean DHP mental health score of at least 12.1 points lower than that of male non-victims (whose JVQ R2 score were 0).

## Discussion

This study is the first from a lower-middle income country to generate robust evidence about the associations between different forms of victimisation, poly-victimisation and health-related quality of life among adolescents. We have shown that different forms of victimisation affected different domains of health-related quality of life and that the most detrimental form of victimisation to adolescents’ HRQoL was different for girls and boys in this sample of Vietnamese students. We have also found that poly-victimisation was associated with poor HRQoL among both sexes.

### Individual forms of victimisation and adolescents’ health related quality of life

Adolescents in Vietnam experience higher rates of any violent victimisation and poly-victimisation than those living in high and upper-middle-income countries such as USA, China or South Africa and lower-middle income countries such as India, El-Salvador and Kenya [24, 36]. Adolescents who had experienced child maltreatment, property victimisation, physical assault, peer/sibling victimisation, sexual victimisation, cyber victimisation and witnessing of family violence had poorer HRQoL on different domains, compared to those who did not. Unexpectedly, adolescents who reported witnessing community violence had better HRQoL in terms of mental and general health, compared to non-witnesses.

#### Child maltreatment and HRQoL

Among this sample of Vietnamese students, almost two in three had experienced child maltreatment during their lifetime. When taking into account other forms of victimisation, among both sexes, victims of child maltreatment had worse HRQoL in terms of mental and general health, lower levels of self-esteem and more symptoms of anxiety than those who did not experience maltreatment. Maltreatment was also associated with poorer social health for girls; and poorer physical health, more symptoms of depression and pain for boys. It was the most detrimental form of victimisation for health-related quality of life among boys and the second most detrimental among girls. This result is consistent with existing evidence from Vietnam of the detrimental impacts of child maltreatment on the mental health and self-esteem of adolescents [4, 40]. We have elucidated and extended previous findings and shown that child maltreatment may affect not only particular aspects of adolescents’ health but also other aspects of their lives including social participation and recreation.

As a result of being ruled by various Chinese empires for over 1000 years, Vietnam has been deeply affected by Confucian ideology [41] in which harsh child discipline is considered to be beneficial for the child. Many parents believe in the maxim “spare the rod, spoil the child” and commonly use harsh physical punishment on their children. Maltreatment is thus prevalent and there is poor recognition in Vietnam of its detrimental impact on the health and wellbeing of children and adolescents. There are no national programs for the prevention of violence against children, including maltreatment. There are also only minimal support services for child victims of violence. This lack of programs and services may have contributed to the poor HRQoL of victims of child maltreatment in this study.

#### Property victimisation and HRQoL

Property victimisation has not previously been investigated among adolescents in Vietnam. Data from this study indicate a high prevalence of property victimisation with as many as six in ten students reporting that they had ever had their belongings stolen or intentionally broken. Female students who experienced this type of victimisation had only significantly poorer HRQoL on the physical, general health and pain domains but not others. In the absence of prior research in this setting we can only speculate on explanations for these findings. It may be that property victimisation is perpetrated by person(s) who are not in a close relationship with the female adolescents; victims may have more easily tolerated having something stolen or broken than others; the belongings which were stolen may have been of unimportance to them; property victimisation may thus only have had an impact on physical health and not on other domains. Compared to female students, male students may not have been affected by experiences of property victimisation because they are physically stronger and therefore, less likely to be a victim of this form of violence.

#### Physical assault and HRQoL

One of the noteworthy findings of this study was the significant association between physical assault and poorer mental, social and general health and more symptoms of anxiety and depression among boys but not girls. These differences may be attributable to the higher prevalence of being physically assaulted among boys compared to girls. The frequency and severity, which was not investigated in this survey, may have also contributed to this result. Boys may experience physical assaults more often and each assault may be more severe than those experienced by girls; a significant association between physical assault and poorer HRQoL may thus have been recorded among boys, but not girls. Others have also found a higher likelihood of boys experiencing physical violence compared to girls [42].

#### Peer/sibling victimisation and HRQoL

Similar to physical assault, peer/sibling victimisation was not associated with any decreased HRQoL among girls, but significantly associated with poorer mental and perceived health, a lower level of self-esteem and more symptoms of anxiety and depression among boys. These results are consistent with previous findings of another study among adolescents in Vietnam [43] which found that bullying was associated with an increased risk of suicidal thoughts among boys, but not girls. Again it is possible that differences in terms frequency and severity of the experiences between boys and girls may explain these results.

#### Sexual victimisation and HRQoL

Among this sample, sexual victimisation was significantly associated with more symptoms of depression among girls and anxiety among boys. This result is consistent with findings reported elsewhere about the associations between sexual violence and mental health problems [44]. It is, however, different from those found in Nguyen et al.’s study about multiple types of maltreatment among Vietnamese students [4]. Nguyen et al. [4] found no significant association between sexual victimisation and symptoms of depression or anxiety among both sexes when taking into account physical and emotional victimisation and neglect. It is noteworthy that sexual victimisation in Nguyen et al.’s study was much more broadly defined than in our study, including not only forced sexual intercourse, inappropriate touching or showing of the genitals, but also forced access to pornographic materials. The wide range of experiences, including some ‘not-as-severe’ types of sexual victimisation, may have explained the non-significant result in Nguyen et al.’s study compared to the significant finding in this study.

#### Cyber victimisation and HRQoL

The Internet and social media has become increasingly popular among Vietnamese people, including adolescents, with the number of Internet users increasing from 200,000 in 2000 to 47.3 million in 2015 and there is now 35 million Facebook users in Vietnam [45]. While this has the benefits of increasing access to information and global participation, it provides another place of potential victimisation. Overall more than one in four adolescents in this study reported having been victimised online.

Cyber victimisation was associated with poorer HRQoL among both girls and boys. It was in fact the most detrimental form of victimisation on HRQoL among girls. This finding is inconsistent with that of others [46]. Chen et al. [46] investigated bullying occurring before and during college among 1452 college students in Taiwan, and found that when physical, relational and verbal bullying were taken into account, cyber bullying occurring before college was associated with better physical HRQoL but not psychological or social HRQoL. The authors proposed that as a result of being victimised online, these students might have reduced the amount of time spent on the Internet and participated in activities which improved their physical health. It is important to note that Chen et al.’s investigation focused on different forms of bullying perpetrated by peers, while our study included other forms of victimisation which may have been perpetrated by others not just peers. It may also be that because the Vietnamese students in our study were younger, the impact of cyber victimisation on their mental health may have been greater than that in Chen et al’s study.

It is possible that our finding also indicates a lack of awareness and poor recognition of cyber victimisation among Vietnamese parents, teachers and the community. Despite the wide use of the Internet, parents may not be aware of cyber victimisation and not have any language to describe or discuss it or to protect their children or to intervene when it occurs. The widespread access to Internet Café or Play-Stations services without any age restriction in Vietnam also means that adolescents are able to access the Internet not only at home, but also outside their house. The amount of time adolescents spend on the Internet and their often unrestricted access to unsafe websites may have contributed to the high prevalence of cyber victimisation found in this study. In addition, similar to other forms of victimisation, there are currently no prevention and intervention programs for cyber victimisation in Vietnam.

#### Witnessing of family violence and HRQoL

When other forms of victimisation were taken into account, female students in this sample who had witnessed violence among their family members had poorer social and general health and lower levels of self-esteem than those who had not witnessed family violence. Male students who had experienced this also had lower levels of self-esteem compared to those who had not witnessed it. This result indicates that for both sexes, family plays an important role in their health-related quality of life and even indirect forms of victimisation such as witnessing of victimisation among family members can affect this outcome. These results were consistent with those found by Kitzmann et al. in their meta-analysis investigating the witnessing of domestic violence and child outcomes [47]. In this review, Kitzmann et al. synthesised the results from 118 studies and concluded that exposure to domestic violence had a significant association with an increased likelihood of mental health, social and academic problems among the child victims compared to non-victims. “Inappropriate attitudes about violence”, “a greater willingness to use violence” and “stronger beliefs about being responsible for their parents’ violence” among the exposed children were discussed as a result of exposure to domestic violence and contribution to their poorer psychological and social functioning [47].

#### Witnessing of community violence and HRQoL

In this study, an unexpected finding was that witnessing violence in the community was associated with better mental health among boys and general health among both sexes. It may be that students who reported witnessing community violence were more likely to be involved in social activities, and be out in the community with friends and family members and thus, more likely to report having witnessed violence in the community. Another explanation may be that it is not uncommon for Vietnamese adolescents to hear or witness neighbourhood violence including robbery, theft and physical fighting. Adolescents may then regard these as a normal part of their daily life and may have developed higher levels of resilience and adaptive skills which may protect them from adverse health impacts. This finding may also indicate that witnessing community violence is a form of indirect victimisation and its impacts may not be as detrimental as other direct forms of victimisation.

Previous studies in high- and upper-middle income countries have also reported that different forms of victimisation affect HRQoL in different ways. Among US adolescent girls, severe physical violence in the form of “hitting, kicking or throwing someone down” by a dating partner has been shown to be associated with poorer physical and mental health, but not self-rated health; forced sex was however associated with poorer physical, mental as well as self-rated health [48]. Among Taiwanese college students, verbal and relational bullying were associated with poorer social HRQoL but not with physical HRQoL while cyber victimisation was associated with better physical HRQoL [46]. Among adults, different types of childhood maltreatment have also been reported to have varied effects on HRQoL [19]. Neglect, psychological, physical and sexual maltreatment were all associated with decreased mental HRQoL; while only psychological and physical maltreatment were associated with reduced physical HRQoL [23]. It may be that certain forms of victimisation result in higher levels of shame and humiliation which lead to poorer mental HRQoL but do not affect the victims’ physical health and therefore, do not show any association with physical HRQoL.

### Poly-victimisation and adolescents’ health related quality of life

The most striking finding of this study is that, compared to non-victims or victims of individual forms of victimisation, poly-victims reported poorer HRQoL across physical, mental, and general health domains; lower levels of self-esteem and increased symptoms of anxiety, depression and pain. This was observable even after taking into account potential confounding factors. These findings are consistent with previous research in upper-middle and high-income countries, which has shown that poly-victimisation during childhood has detrimental impacts on HRQoL during adolescence [23], and adulthood [19, 49].

The associations between individual forms of victimisation and HRQoL were not as large as those between poly-victimisation and HRQoL. Individual forms of victimisation may not be significantly associated with HRQoL when their relationships with HRQoL are examined simultaneously. Investigations limited to individual forms of victimisation may therefore result in misleading conclusions about the significant associations between these forms of victimisation and HRQoL when in fact, the observed associations are attributable to the cumulative exposure to poly-victimisation, or to other forms of victimisation co-occurring with this form which, if not ascertained, are invisible.

Overall these data provide strong evidence that poly-victimisation is a superior construct to individual forms of victimisation in research in understanding the impact of violence against children and adolescents and their implications for policies and programs. Young people live in a wider context of their family, school, community and society. They experience different forms of victimisation, including not only child maltreatment, but also peer victimisation, property victimisation, witnessing of community violence and cyber victimisation. Investigations of violence against young people, which are limited to only child maltreatment or individual forms of victimisation, overlook other forms of victimisation and the cumulative impact of exposure to poly-victimisation, which we have shown to have the most detrimental effects on adolescents’ HRQoL. Price-Robertson et al. concluded in a comparison of the explanatory power of different conceptual frameworks of violence among Australian young adults that poly-victimisation enabled the capture of a “greater range of adversity” and was associated with “a greater number of problematic outcomes in early adulthood” [50]. Compared to those who experienced only maltreatment or bullying, those who experienced poly-victimisation (both maltreatment and bullying) were significantly more likely to be depressed or anxious or to experience chronic health problems or to have disturbed conduct.

### Gender inequality in health-related quality of life

Compared to another sample of 1408 adolescents aged 12–19 years in Vietnam [26], students in this sample had poorer physical, mental and general health, but had better social and perceived health. Girls in this sample had lower levels of self-esteem compared to those in Hanh et al.’s study, but boys had higher levels of self-esteem compared to those in Hanh et al.’s. Recruitment differences between the two studies may explain this result. Adolescents in our study were from Hanoi, the capital city in the North of Vietnam, while those in Hanh et al.’s were from Ho Chi Minh City, which is in the South of Vietnam and is the business and economic capital of the country. Better economic development may have contributed to the better physical, mental and general health among adolescents in Ho Chi Minh City, compared to those in Hanoi. However, the traditions of a close community and extended family and higher levels of optimism in the North may have meant that adolescents living in the North were more likely to meet friends and relatives than those in the South; thus having better social and perceived health. Differences in terms of risk factors, including exposure to violence, may have also contributed to the result.

These data have also revealed significant gender inequality in HRQoL in Vietnam: Vietnamese adolescent girls had significantly poorer HRQoL on all domains, except disability, compared to boys. In Vietnamese families, boy-children are often preferred to girl children because they are believed to maintain the family name and line. Girls are expected to do more household chores and thus, have fewer educational opportunities [41, 51]. When the family is poor and cannot afford tuition fees, it is often the female child who has to leave school while the male child continues their education. Vietnamese girls are more likely to experience neglect and emotional abuse than boys [4]. In Hanh et al.’s validation study of the DHP-A [26] among a sample of 1408 adolescents aged 12–19 years in Vietnam, significantly lower scores on all domains of the DHP-A, expect disability, were reported among female students, compared to males. It may be that this pervasive devaluing of girls is reflected in the generally worse HRQoL among adolescent girls compared to boys found in this study.

### Strengths and limitations

The data in this study were drawn from a survey, which included international standardised measures, of a large, systematically-recruited sample with a high response rate. Diverse forms of victimisation, including some which had never been investigated in Vietnam, were examined. This has enabled us to provide a detailed description of the associations between experiences of violence and Vietnamese adolescents’ health-related quality of life. We believe that the findings can be generalised with considerable confidence to high school students in Vietnam; but acknowledge that they do not describe the experiences of adolescents who are not attending school, are members of ethnic minority groups or are living in remote areas of the country. The use of cross-sectional data also affects the ability to make conclusions about causal-relationships between experiences of violence and poorer HRQoL.

## Conclusions

Vietnamese adolescents experience violent victimisation and poly-victimisation at higher rates than adolescents in high-income countries [36]. Victimisation is associated with poor HRQoL, an important indicator of adolescents’ health and wellbeing. Health and wellbeing in adolescence predicts these in adulthood [52, 53], and are associated with social and economic participation and therefore, are of relevance to the nation’s development. There is currently a lack of awareness about the prevalence and impact of violence, including poly-victimisation, on the health and wellbeing of adolescents in Vietnam. These data indicate that prevention of violence against children and adolescents in Vietnam warrants prioritisation so that their own development and ultimately that of the country are optimised. The findings of this study have important implications for policy, programs and future research.

There is currently only minimal recognition of violence against children and adolescents at the policy level in Vietnam. The Law on Domestic Violence prohibits intimate partner violence (IPV) in families; perpetrators of IPV may be subjected to certain fines. Many female victims of domestic violence thus do not disclose information about such incidents, being afraid of financial sanctions imposed on their husband, which, in the end, may affect the financial situation of the whole family. The Law on Care and Protection of Vietnamese children acknowledges the importance of prevention of violence against them and prohibits any acts of violence, abuse or trafficking of children in Vietnam; however, the Law focuses mostly on child maltreatment within families and there is not yet any recognition of the wider aspects of victimisation and poly-victimisation. Approaches taken from other countries, like Australia, to address this include prohibition of any corporal punishment at school or “unreasonable” corporal punishment by parents [54]; and mandatory reporting of suspected child abuse and neglect [55]. Data from this study suggest that Vietnam should investigate the application of these approaches in order to prevent violence against children.

In terms of programs, a free Helpline is available to support Vietnamese children. However, to our knowledge, there has been no official evaluation of its effectiveness. The high prevalence of poly-victimisation found in our study suggests that this Helpline alone is not sufficient to prevent violence or support victims of violence. There are currently no other national programs for the prevention of violence against children or interventions for child victims of violence. Development of additional programs, in which gender differences are considered and gender inequality is addressed, is important. Evaluating and adapting existing interventions, such as school-based educational programs [56] and positive parenting practice [57, 58], to the circumstances of Vietnam may be helpful. Unlike in high income countries where there are social workers and specific child protection authorities; in Vietnam, child protection mostly relies on parents, teachers, the police and Youth and Women Unions at local communes. Educating the community about violence via existing networks, including Youth Unions or Women Unions available at each commune level, may also be beneficial.

In terms of directions for future research, the results highlight the need for comprehensive investigations of multiple forms of violence among adolescents. Similar to the conclusions of others [8, 34, 50], using poly-victimisation as a framework for research about violence against children and adolescents is recommended; investigations focusing on an individual form of violence should take into account other forms.

## Additional files

Additional file 1: Table S1. Items used to create the ten DHP-A subscales. (DOCX 13 kb)
Additional file 2: Table S2. Details of JVQ R2 items used to create eight aggregate victimisation modules. (DOCX 16 kb)
Additional file 3: Table S3. Correlations between eight forms of victimisation examined. (DOCX 16 kb)
Additional file 4: Table S4. Relationships between different forms of victimisation and six health domains of the DHP-A among Vietnamese high school students – bivariate analyses^a,b^ (each form of victimisation was entered into separate models). (DOCX 17 kb)
Additional file 5: Table S5. Relationships between different forms of victimisation and four dysfunction domains of the DHP-A among Vietnamese high school students – bivariate analyses^a, b^ (each form of victimisation was entered into separate models). (DOCX 15 kb)

## Abbreviations

CI: Confidence interval
DHP-A: Duke Health Profile Adolescent Version
HRQoL: Health-related quality of life
JVQ R2: Juvenile Victimisation Questionnaire Revised 2
