# Supplementary material for: Victimisation, poly-victimisation and health-related quality of life among high school students in Vietnam: a cross-sectional survey
Source: Health Qual Life Outcomes. 2016 Nov 4;14:155. doi: 10.1186/s12955-016-0558-8 (PMC5097374; doi:10.1186/s12955-016-0558-8)
Supplement: Additional file 1: Table S1. — Items used to create the ten DHP-A subscales. (DOCX 13 kb) [file 12955_2016_558_MOESM1_ESM.docx]

**Supplementary Table 1. Items used to create the ten DHP-A subscales**

| DHP-A items | Subscale |
| --- | --- |
| 1. I am satisfied with myself | Mental health, Self-esteem |
| 2. I am not an easy person to get along with | Social health, Self-esteem, Anxiety |
| 3. I am basically a healthy person | Perceived health |
| 4. I give up too easily | Mental health, Self-esteem, Depression |
| 5. I have difficulty concentrating | Mental health, Anxiety, Depression |
| 6. I am happy with my family relationships | Social health, Self-esteem |
| 7. I feel at ease being around people | Social health, Self-esteem, Anxiety |
| TODAY: would you have any physical trouble or difficulty…? |  |
| 8. walking up a flight of stairs | Physical health |
| 9. Running the length of 100 meters or the distance between three light poles | Physical health |
| DURING LAST WEEK: How much trouble have you had with? |  |
| 10. sleeping | Physical health, Anxiety, Depression |
| 11. hurting or aching in any part of your body | Physical health, Pain |
| 12. getting tired easily | Physical health, Anxiety, Depression |
| 13. feeling depressed or sad | Mental health, Depression |
| 14. nervousness | Mental health, Anxiety |
| DURING LAST WEEK: How often did you? |  |
| 15. Socialise with other people (talk or visit with friends or relatives) | Social health |
| 16. Take part in social, religious or recreation activities (meetings, church, pagoda, movies, sports, parties) | Social health |
| DURING LAST WEEK: How often did you |  |
| 17. Stay in your house or hospital because of sickness, injury, or other health problems | Disability |
