# Supplementary material for: Victimisation, poly-victimisation and health-related quality of life among high school students in Vietnam: a cross-sectional survey
Source: Health Qual Life Outcomes. 2016 Nov 4;14:155. doi: 10.1186/s12955-016-0558-8 (PMC5097374; doi:10.1186/s12955-016-0558-8)
Supplement: Additional file 2: Table S2. — Details of JVQ R2 items used to create eight aggregate victimisation modules. (DOCX 16 kb) [file 12955_2016_558_MOESM2_ESM.docx]

**Supplementary Table 2. Details of JVQ R2 items used to create eight aggregate victimisation modules**

| Victimisation form | Aggregate module |
| --- | --- |
| Conventional Crime |  |
| Robbery: At any time in your life, did anyone use force to take something away from you that you were carrying or wearing? | Property victimisation |
| Personal theft: At any time in your life, did anyone steal something from you and never give it back? Things like a backpack, money, watch, clothing, bike, stereo, or anything else? |  |
| Vandalism: At any time in your life, did anyone break or ruin any of your things on purpose? |  |
| Assault with weapon: Sometimes people are attacked with sticks, rocks, guns, knives, or other things that would hurt. At any time in your life, did anyone hit or attack you on purpose with an object or weapon? Somewhere like: at home, at school, at a store, in a car, on the street, or anywhere else? | Physical assault |
| Assault without weapon: At any time in your life, did anyone hit or attack you without using an object or weapon? |  |
| Attempted assault: At any time in your life, did someone start to attack you, but for some reason, it didn't happen? For example, someone helped you or you got away? |  |
| Kidnapping: When a person is kidnapped, it means they were made to go somewhere, like into a car, by someone who they thought might hurt them. At any time in your life, did anyone try to kidnap you? |  |
| Bias attack: At any time in your life, have you been hit or attacked because of your skin colour, religion, or where your family comes from? Because of a physical problem you have? Or because someone said you were gay? |  |
| Child maltreatment |  |
| Physical abuse by caregiver: Not including spanking on your bottom, at any time in your life, has a grown-up in your life hit, beat, kick, or physically hurt you in any way? | Maltreatment |
| Emotional abuse: At any time in your life, did you get scared or feel really bad because grown-ups in your life called you names, said mean things to you, or said they didn't want you? |  |
| Neglect: When someone is neglected, it means that the grown-ups in their life didn't take care of them the way they should. They might not give them enough food, take them to the doctor when they are sick, or make sure they have a safe place to stay. At any time in your life, were you neglected? |  |
| Family abduction: Sometimes a family fights over where a child should live. At any time in your life, did a parent take, keep, or hide you to stop you from being with another parent? |  |
| Peer and sibling victimisation |  |
| Gang or group assault: Sometimes groups of kids or gangs attack people. At any time in your life, did a group of kids or a gang hit, jump, or attack you? | Peer/sibling victimisation |
| Peer or sibling assault: At any time in your life, did any kid, even a brother or sister, hit you? Somewhere like: at home, at school, out playing, in a store, or anywhere else? |  |
| Nonsexual Genital Assault: At any time in your life, did any kids tried to hurt your private parts on purpose by hitting or kicking you there? |  |
| Physical Intimidation by peers: At any time in your life, did any kids, even a brother or sister, pick on you by chasing you or grabbing you or by making you do something you didn't want to do? |  |
| Relational aggression by peers: At any time in your life, did you get scared or feel really bad because kids were calling you names, saying mean things to you, or saying they didn't want you around? |  |
| Dating violence: At any time in your life, did a boyfriend or girlfriend or anyone you went on a date with slap or hit you?  Experienced dating violence by a boy/girlfriend |  |
| Sexual victimisation |  |
| Sexual assault by known adult: At any time in your life, did a grown-up you know touch your private parts when they shouldn't have or make you touch their private parts? Or did a grown-up you know force you to have sex? | Sexual victimisation |
| Sexual assault by unknown adult: At any time in your life, did a grown-up you did NOT know touch your private parts when they shouldn't have, make you touch their private parts or force you to have sex? |  |
| Sexual assault by peer/ sibling: Now think about kids your age, like from school, a boyfriend or girlfriend, or even a brother or sister. At any time in your life, did another child or teen make you do sexual things? |  |
| Forced sex: At any time in your life, did anyone try to force you to have sex; that is, sexual intercourse of any kind, even if it didn't happen? |  |
| Flashing/ sexual exposure: At any time in your life, did anyone make you look at their private parts by using force or surprise, or by "flashing" you? |  |
| Verbal sexual harassment: At any time in your life, did anyone hurt your feelings by saying or writing something sexual about you or your body? |  |
| Witnessing and indirect victimisation |  |
| Witness to domestic violence: At any time in your life, did you SEE a parent get pushed, slapped, hit, punched, or beat up by another parent, or their boyfriend or girlfriend? | Witnessing of family violence |
| Witness to parent assault of sibling: At any time in your life, did you SEE a parent hit, beat, kick, or physically hurt your brothers or sisters, not including a spanking on the bottom? |  |
| Witness to assault with weapon: At any time in your life, in real life, did you SEE anyone get attacked on purpose WITH a stick, rock, gun, knife, or other thing that would hurt? Somewhere like: at home, at school, at a store, in a car, on the street, or anywhere else? | Witnessing of community violence |
| Witness to assault without weapon: At any time in your life, in real life, did you SEE anyone get attacked or hit on purpose WITHOUT using a stick, rock, gun, knife, or something that would hurt? |  |
| Burglary of family household: At any time in your life, did anyone steal something from your house that belongs to your family or someone you live with? Things like a TV, stereo, car, or anything else? |  |
| Murder of family member or friend: When a person is murdered, it means someone killed them on purpose. At any time in your life, was anyone close to you murdered, like a friend, neighbour, or someone in your family? |  |
| Exposure to random shootings, terrorism or riots: At any time in your life, were you in any place in real life where you could see or hear people being shot, bombs going off, or street riots? |  |
| Exposure to war or ethnic conflict: At any time in your life, were you in the middle of a war where you could hear real fighting with guns or bombs? |  |
| Family violence and abuse |  |
| Parental Displaced aggression: At any time in your life, did one of your parents, because of an argument, break or ruin anything belonging to another parent, punch the wall, or throw something? | Witnessing of family violence |
| Other family violence exposure: Now we want to ask you about fights between any grown-ups and teens, not just between your parents. At any time in your life, did any grown-up or teen who lives with you push, hit, or beat up someone else who lives with you, like a parent, brother, grandparent, or other relative? |  |
| Internet harassment: Has anyone ever used the Internet to bother or harass you or to spread mean words or pictures about you? | Cyber victimisation |
