# Supplementary material for: Victimisation, poly-victimisation and health-related quality of life among high school students in Vietnam: a cross-sectional survey
Source: Health Qual Life Outcomes. 2016 Nov 4;14:155. doi: 10.1186/s12955-016-0558-8 (PMC5097374; doi:10.1186/s12955-016-0558-8)
Supplement: Additional file 3: Table S3. — Correlations between eight forms of victimisation examined. (DOCX 16 kb) [file 12955_2016_558_MOESM3_ESM.docx]

**Supplementary Table 3. Correlations between eight forms of victimisation examined.**

|  | 1 | 2 | 3 | 4 | 5 | 6 | 7 | 8 |
| --- | --- | --- | --- | --- | --- | --- | --- | --- |
| 1. Property victimisation | 1.00 |  |  |  |  |  |  |  |
| 2. Physical Assault | 0.35 | 1.00 |  |  |  |  |  |  |
| 3. Maltreatment | 0.25 | 0.31 | 1.00 |  |  |  |  |  |
| 4. Peer/ sibling victimisation | 0.32 | 0.39 | 0.33 | 1.00 |  |  |  |  |
| 5. Sexual victimisation | 0.23 | 0.24 | 0.23 | 0.23 | 1.00 |  |  |  |
| 6. Witnessing of family violence | 0.27 | 0.24 | 0.36 | 0.27 | 0.25 | 1.00 |  |  |
| 7. Witnessing of community violence | 0.29 | 0.28 | 0.24 | 0.28 | 0.22 | 0.33 | 1.00 |  |
| 8. Cyber victimisation | 0.20 | 0.21 | 0.19 | 0.26 | 0.25 | 0.17 | 0.20 | 1.00 |
