# Supplementary material for: Victimisation, poly-victimisation and health-related quality of life among high school students in Vietnam: a cross-sectional survey
Source: Health Qual Life Outcomes. 2016 Nov 4;14:155. doi: 10.1186/s12955-016-0558-8 (PMC5097374; doi:10.1186/s12955-016-0558-8)
Supplement: Additional file 4: Table S4. — Relationships between different forms of victimisation and six health domains of the DHP-A among Vietnamese high school students – bivariate analysesa,b (each form of victimisation was entered into separate models). (DOCX 17 kb) [file 12955_2016_558_MOESM4_ESM.docx]

**Supplementary Table 4. Relationships between different forms of victimisation and six health domains of the DHP-A among Vietnamese high school students – bivariate analyses ^a,b^ (each form of victimisation was entered into separate models)**

|  | Physical health | | Mental health | | Social health | | Perceived health | | General health | | Self esteem | |
| --- | --- | --- | --- | --- | --- | --- | --- | --- | --- | --- | --- | --- |
|  | β (95%CI) | | β (95%CI) | | β (95%CI) | | β (95%CI) | | β (95%CI) | | β (95%CI) | |
|  | Females | Males | Females | Males | Females | Males | Females | Males | Females | Males | Females | Males |
| Any property victimisation (yes vs no) | **-7.1**  **(-10.1; -4.0)** | **-3.7**  **(-6.3; -1.1)** | **-3.5**  **(-6.9; -0.1)** | **-6.7**  **(-9.8; -3.7)** | -1.3  (-4.5; 1.8) | **-4.0**  **(-6.6; -1.3)** | -2.8  (-8.3; 2.8) | -2.2  (-7.1; 2.7) | **-4.0**  **(-6.2; -1.7)** | **-4.8**  **(-6.8; -2.8)** | -2.9  (-6.2; 0.3) | **-6.3**  **(-8.9; -3.6)** |
| Any physical assault (yes vs no) | -2.7  (-5.6; 0.1) | **-3.7**  **(-6.3; -1.1)** | **-5.0**  **(-8.1; -1.9)** | **-7.8**  **(-10.8;-4.8)** | **-3.1**  **(-6.0; -0.2)** | **-5.3**  **(-7.9; -2.6)** | 2.2  (-2.9; 7.4) | -2.4  (-7.3; 2.4) | **-3.6**  **(-5.7; -1.5)** | **-5.6**  **(-7.5; -3.6)** | **-4.8**  **(-7.8; -1.9)** | **-6.4**  **(-9.1; -3.8)** |
| Any child maltreatment (yes vs no) | -**3.3**  **(-6.4; -0.1)** | **-6.2**  **(-8.8; -3.6)** | **-7.0**  **(-10.4; -3.6)** | **-7.6**  **(-10.6; -4.5)** | **-6.6**  **(-9.8; -3.5)** | **-4.5**  **(-7.2; -1.8)** | 2.4  (-3.2; 8.1) | -1.9  (-6.9; 3.0) | **-5.6**  **(-7.9; -3.4)** | **-6.1**  **(-8.1; -4.1)** | **-8.6**  **(-11.8; -5.4)** | **-7.6**  **(-10.3; -5.0)** |
| Any peer or sibling victimisation (yes vs no) | -2.9  (-5.8; 0.04) | **-4.2**  **(-6.8; -1.6)** | **-3.7**  **(-6.9; -0.5)** | **-8.9**  **(-11.9; -5.9)** | -2.5  (-5.5; 0.5) | **-4.3**  **(-6.9; -1.6)** | 4.4  (-0.9; 9.7) | **-5.4**  **(-10.2; -0.6)** | **-3.0**  **(-5.2; -0.9)** | **-5.8**  **(-7.7; -3.8)** | **-3.7**  **(-6.8; -0.7)** | **-7.7**  **(-10.3; -5.0)** |
| Any sexual victimisation (yes vs no) | -1.8  (-4.8; 1.2) | -2.7  (-5.8; 0.5) | **-3.8**  **(-7.2; -0.5)** | **-6.9**  **(-10.7; -3.2)** | -1.3  (-4.4; 1.8) | **-3.7**  **(-7.0; -0.4)** | -2.0  (-7.5; 3.4) | 2.9  (-3.1; 8.8) | **-2.3**  **(-4.5; -0.1)** | **-4.4**  **(-6.9; -2.0)** | **-3.2**  **(-6.3; -0.03)** | **-5.8**  **(-9.0; -2.5)** |
| Any witnessing of family violence (yes vs no) | **-3.4**  **(-6.5; -0.3)** | **-2.8**  **(-5.4; -0.1)** | **-5.4**  **(-8.8; -2.0)** | **-4.8**  **(-7.9; -1.7)** | **-4.5**  **(-7.7; -1.3)** | **-4.0**  **(-6.7; -1.2)** | -0.8  (-6.5; 4.9) | -0.4  (-5.4; 4.6) | **-4.4**  **(-6.7; -2.1)** | **-3.8**  **(-5.9; -1.8)** | **-8.4**  **(-11.6; -5.1)** | **-6.2**  **(-8.9; -3.4)** |
| Any witnessing of community violence (yes vs no) | -1.0  (-4.7; 2.8) | -0.8  (-3.8; 2.1) | -1.7  (-5.8; 2.4) | -1.0  (-4.6; 2.5) | 3.4  (-0.4; 7.2) | 0.01  (-3.1; 3.1) | 3.1  (-3.6; 9.9) | 0.5  (-5.1; 6.1) | 0.3  (-2.5; 3.0) | -0.6  (-2.9; 1.7) | -3.0  (-6.9; 0.9) | -1.7  (-4.8; 1.4) |
| Any cyber victimisation (yes vs no) | **-7.7**  **(-10.8; -4.5)** | **-3.5**  **(-6.3; -0.7)** | **-8.7**  **(-12.1; -5.2)** | **-7.3**  **(-10.6; -4.0)** | **-3.5**  **(-6.7; -0.2)** | -0.9  (-3.8; 2.0) | -3.5  (-9.2; 2.3) | -2.4  (-7.7; 2.9) | **-6.6**  **(-8.9; -4.3)** | **-3.9**  **(-6.1; -1.7)** | **-5.5**  **(-8.8; -2.2)** | **-5.0**  **(-8.0; -2.1)** |
| ^a^ Model adjusted for age, rural/ urban residence, family composition, socio-economic status, presence of a chronic disease or disability, school type and number of adverse life events experienced.  ^b^ Significant results are in bold | | | | | | | | | | | | |
