# Supplementary material for: Victimisation, poly-victimisation and health-related quality of life among high school students in Vietnam: a cross-sectional survey
Source: Health Qual Life Outcomes. 2016 Nov 4;14:155. doi: 10.1186/s12955-016-0558-8 (PMC5097374; doi:10.1186/s12955-016-0558-8)
Supplement: Additional file 5: Table S5. — Relationships between different forms of victimisation and four dysfunction domains of the DHP-A among Vietnamese high school students – bivariate analysesa, b (each form of victimisation was entered into separate models). (DOCX 15 kb) [file 12955_2016_558_MOESM5_ESM.docx]

**Supplementary Table 5. Relationships between different forms of victimisation and four dysfunction domains of the DHP-A among Vietnamese high school students – bivariate analyses ^a, b^ (each form of victimisation was entered into separate models)**

|  | Anxiety | | Depression | | Pain | | Disability | |
| --- | --- | --- | --- | --- | --- | --- | --- | --- |
|  | **Adjusted β (95%CI)** | | **Adjusted β (95%CI)** | | **Adjusted β (95%CI)** | | **Adjusted β (95%CI)** | |
|  | **Females** | **Males** | **Females** | **Males** | **Females** | **Males** | **Females** | **Males** |
| Any property victimisation (yes vs no) | **4.5**  **(1.6; 7.3)** | **6.4**  **(3.8; 9.0)** | **4.8**  **(1.5; 8.1)** | **5.5**  **(2.5; 8.6)** | **10.2**  **(5.2; 15.2)** | **5.2**  **(0.9; 9.5)** | 2.5  (-1.1; 6.0) | -1.4  (-5.1; 2.4) |
| Any physical assault (yes vs no) | **4.7**  **(2.1; 7.3)** | **7.3**  **(4.7; 9.9)** | **5.6**  **(2.5; 8.6)** | **7.7**  **(4.6; 10.7)** | 4.5  (-0.2; 9.1) | **5.8**  **(1.4; 10.1)** | -0.2  (-3.5; 3.2) | 3.1  (-0.7; 6.8) |
| Any child maltreatment (yes vs no) | **6.3**  **(3.4; 9.1)** | **7.1**  **(4.4; 9.7)** | **5.4**  **(2.0; 8.7)** | **7.5**  **(4.4; 10.6)** | **6.6**  **(1.5; 11.7)** | **8.6**  **(4.2; 13.0)** | -2.8  (-6.4; 0.8) | 2.3  (-1.5; 6.2) |
| Any peer or sibling victimisation (yes vs no) | **4.4**  **(1.7; 7.0)** | **7.4**  **(4.7; 10.0)** | **3.3**  **(0.1; 6.4)** | **8.0**  **(4.9; 11.0)** | **6.0**  **(1.3; 10.7)** | **7.1**  **(2.7; 11.5)** | 1.2  (-2.2; 4.6) | 2.1  (-1.7; 5.9) |
| Any sexual victimisation (yes vs no) | **3.5**  **(0.7; 6.3)** | **6.7**  **(3.5; 10.0)** | **5.7**  **(2.4; 8.9)** | **7.0**  **(3.2; 10.7)** | 2.2  (-2.8; 7.1) | **6.7**  **(1.3; 12.1)** | 0.6  (-2.9; 4.0) | -2.6  (-7.3; 2.1) |
| Any witnessing of family violence (yes vs no) | **4.3**  **(1.4; 7.2)** | **4.2**  **(1.5; 6.9)** | **4.6**  **(1.3; 8.0)** | **4.0**  **(0.8; 7.1)** | 5.0  (-0.1; 10.1) | **5.2**  **(0.7; 9.6)** | -1.4  (-5.0; 2.2) | -0.6  (-4.5; 3.2) |
| Any witnessing of community violence (yes vs no) | 0.05  (-3.4; 3.5) | 1.9  (-1.1; 5.0) | 0.09  (-3.9; 4.1) | 0.6  (-3.0; 4.1) | **7.3**  **(1.2; 13.4)** | 3.6  (-1.5; 8.7) | 0.2  (-4.2; 4.5) | -1.2  (-5.5; 3.1) |
| Any cyber victimisation (yes vs no) | **7.1**  **(4.2; 10.0)** | **5.1**  **(2.2; 8.0)** | **8.0**  **(4.6; 11.4)** | **7.5**  **(4.2; 10.9)** | **7.8**  **(2.5; 13.1)** | **6.2**  **(1.4; 11.0)** | **3.9**  **(0.3; 7.6)** | -0.3  (-4.4; 3.8) |
| ^a^ Model adjusted for age, rural/ urban residence, family composition, socio-economic status, presence of a chronic disease or disability, school type and number of adverse life events experienced.  ^b^ Significant results are in bold. | | | | | | | | |
